# Ruminal pH sensing for monitoring volatile fatty acid concentrations in response to short-term dietary disruption

**DOI:** 10.3168/jdsc.2023-0409

**Published:** 2023-11-17

**Authors:** K. Amirault, R. Wright, S. Sujani, B.R. dos Reis, J. Osorio, T. Fernandes, R.R. White

**Affiliations:** School of Animal Sciences, Virginia Tech, Blacksburg, VA 24073

## Abstract

The purpose of this study was to investigate the potential of using ruminal pH measurements to track time-series ruminal volatile fatty acid (VFA) concentrations occurring in response to short-term dietary disruption. Four ruminally cannulated dry Holstein dairy cows were individually housed and assigned to 4 treatments in a Latin square design. Treatments differing in forage-to-concentrate (F:C) ratio (100:0 to 55:45) were used because they were expected to result in large differences in VFA concentration, over which the relationships between pH and VFA could be robustly evaluated. Each sampling period lasted 36 h. Animals were removed from pasture and fasted for 24 h, after which time they were fed their treatment ration for 2 h and sampled for rumen fluid hourly for 12 h. Rumen fluid samples were analyzed immediately for pH, frozen, and subsequently analyzed for VFA concentrations using gas chromatography. Animals were returned to pasture for 7 d between sampling periods. To confirm that the short-term dietary disruptions resulted in expected variation in VFA concentrations, mean VFA concentrations during each animal period (n = 16) were analyzed using a linear mixed effects model with fixed (linear and quadratic) effects for F:C ratio and random effects for animal and period. Results indicated significant changes in VFA concentration across F:C ratio, but no significant shifts in VFA molar proportions, perhaps due to the short-term nature of the feeding protocol. To explore opportunity to use pH measurements to explain variability in VFA concentrations in real time across dietary conditions, a linear mixed-effect model was used to link the time-series measurements (n = 207). The VFA concentrations were analyzed with linear mixed effect models using linear and quadratic terms for pH, and random effects for animal and period. These models had poor accuracy, with residual error variance ranging from 21% to 38%, and residuals patterning significantly with F:C ratio. The data suggest that pH may lack reliability for VFA prediction in short-term feeding scenarios differing considerably in F:C ratio.

## Highlights

• Sensor technologies do not currently monitor ruminal VFA concentrations.
• pH measurements relate to VFA, but relationships are diet dependent.
• pH measurements have poor accuracy in predicting VFA.

## Graphical Abstract

**Figure:**
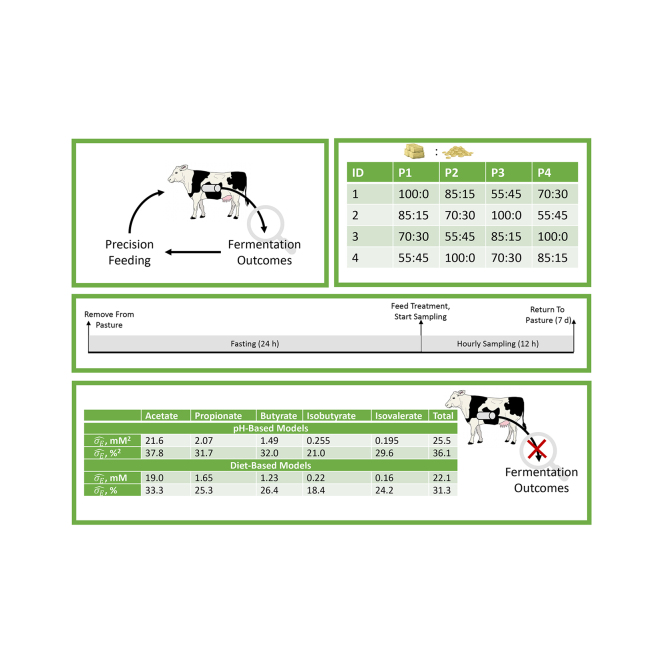
**Summary** The synthesis of volatile fatty acids (VFA), a primary product of microbial fermentation that provides a crucial source of energy for the animal, is driven primarily by diet characteristics. Exploration of commercially available sensors to estimate ruminal VFA concentrations across diets could drastically change how ration-formulation decisions are made. This study investigated the opportunity to leverage ruminal pH to track VFA concentrations in the rumen in response to short-term dietary disruption. Although significant, pH measurements have poor accuracy in predicting VFA, and prediction errors pattern with dietary forage to concentrate ratio.

Although precision feeding presents a tremendous opportunity to enhance a variety of productivity-related objectives on dairy operations, decision-making tools to support precision feeding have limited capacity for real-time monitoring of digestion and metabolism (Price et al., 2021; Souza and White, 2021). To address this limitation, monitoring tools that are capable of representing digestion and metabolism in real time and in a manner sensitive to and robust across short-term feeding changes are needed. Ruminal pH measurements are widely used as indicators of rumen health and function, and may provide use in this context due to their ability to rapidly detect short-term changes in rumen conditions such as those occurring during the onset of subacute ruminal acidosis (Enemark, 2008; Sato, 2016). Early ruminal pH measurements were collected in vitro (Monroe and Perkins, 1939); however, in vivo pH measurement techniques were developed (Smith, 1941) after identifying that pH measurements changed with sample exposure to air. Ruminal pH monitoring technologies then came into use in the 1950s (Lampila, 1955), and these technologies have been refined over time to yield indwelling pH sensors (Penner et al., 2006) and boluses (Sato et al., 2012) used today.

Although rumen pH sensors could be a valuable tool supporting physiological monitoring needed to enable more targeted precision feeding, they have not been investigated for this purpose. A critical initial question toward this longer-term goal centers on whether rumen pH monitoring can be used to represent energy-yielding end products produced through fermentation (i.e., VFA). It is plausible that pH could be used as a representation of VFA concentrations due to the physiological linkages among these factors. For example, changes in ruminal pH can directly affect the microbial metabolism, thereby influencing VFA production in the rumen (Dijkstra et al., 2012). Conversely, the production of VFA can also influence ruminal pH (Penner, 2014). As such, we hypothesized that ruminal pH dynamics may be sensitive enough to provide real-time indication of the end products present in the rumen in response to short-term dietary disturbances such as those that might occur during precision feeding. Furthermore, we expected that the relationships between pH and VFA concentrations would be robust across a range of dietary interventions, meaning that VFA could be predicted from pH without the need for diet-based adjustment to the prediction functions.

The aim of this study is to evaluate the associations among time-series ruminal VFA concentrations and pH in response to short-term diet disruption. As a secondary goal, we sought to compare the variability in VFA measurements explained by pH with variability that could be explained by diet and time postfeeding to contextualize the value of estimating VFA concentrations from diet parameters or rumen pH.

All animals sourced in this study belonged to Virginia Polytechnic and State University. All procedures with animals were performed in accordance with the protocols approved by the Institutional Animal and Care and Use Committee at Virginia Polytechnic and State University (IACUC #22–074).

Four ruminally cannulated dry Holstein dairy cows at maintenance were exposed to each of 4 forage-to-concentrate (**F:C**) ratios through 4 experimental periods in a 4 × 4 Latin square design. This design yielded 16 animal periods, which constituted the experimental unit for comparison of ruminal conditions, and 208 observations available for comparison of pH and VFA concentrations. All animals were housed at the Virginia Tech Dairy for the duration of the study. Cows were maintained on cool season grass pasture before the start of the experiment and for at least 7 d between sampling periods. Sampling periods lasted 36 h. Animals were removed from pasture, fasted for 24 h, fed their treatment ration, and monitored for 12 h. During this 36-h period, animals were housed in individual pens with ad libitum water. Animals were allowed access to treatment diets from 0600 to 0800 h after completing the fasting period, meaning that substrate delivery to the rumen was confined to a single, large meal to facilitate time-series monitoring. Before feeding and after the 24 h fast, a baseline rumen fluid sample was collected, with subsequent samples obtained hourly for 12 h. This sampling protocol resulted in a total of 13 samples per period per animal. Obtained samples were analyzed for pH directly following sampling, and were then frozen and stored at −20°C until analysis for VFA concentrations. Ruminal pH was measured directly on samples used for VFA determination to avoid potential confounding effects of sampling location within the rumen. As such, these data best represent alignment between pH and VFA within a single fluid sample, rather than pH observations that might be obtained from bolus or sensor technologies which often sink into the reticulum and may not reflect pH from the same fluid used for VFA determination. This experimental choice was made to maximize the likelihood of detecting a relationship between pH and VFA.

Diets were formulated to meet or exceed the energy and protein requirements of dry cows using the NASEM (2021) model. Treatments varying in concentrate (corn grain and soybean meal) inclusion to yield F:C ratios ranging from 100:0 to 55:45 (Table 1) were selected because they were expected to generate considerable differences in total VFA production and in profiles of VFA produced based on the broad body of literature previously studying ruminal conditions on similar diets after dietary adaptation. Due to the short feeding duration used in this study, we were not certain that short-term changes rumen conditions would be of similar magnitude and direction to the shifts revealed from long-term feeding studies; therefore, the conservative selection of treatments was also an attempt to maximize likelihood of creating differences in VFA concentrations from short-term diet shifts. Treatments reflected a linear increase in concentrate inclusion percentage and the NASEM (2021)-formulated dietary metabolizable energy, net energy, and ruminally degradable protein for these diets, as well as the chemical composition and measured feedstuff inclusion, are presented in Table 1. The range of F:C ratios was determined based on the ranges typically used in dairy rations, to reflect the most extreme changes in rumen conditions that might be expected. Testing these F:C ratios in the short term allowed for conservative assessment of whether pH sensing would be sensitive enough to detect changes in rumen conditions that might eventually be used to support precision feeding approaches.

**Table 1:** Chemical composition and nutrient inclusion for each treatment diet

Item	Treatment diet^1^			
	100:0	85:15	70:30	55:45
Diet composition, kg (as fed)				
Grass hay	12.34	10.48	8.66	6.80
Cracked corn	—	0.64	1.22	1.86
Soybean meal	—	1.22	2.45	3.72
NASEM (2021) formulated composition				
Diet digestible energy, Mcal/kg	2.81	2.88	2.97	3.10
Diet ME, Mcal/kg	2.48	2.45	2.45	2.47
Diet RDP, % DM	5.57	8.61	11.7	14.87
				
Ingredient composition,^2^ % DM	Hay	CG	SBM	
% DM	85.2	84.1	87	
CP	8.7	9.1	52.9	
Fat	2.2	4.2	2.3	
NDF	70.9	10.3	8.9	
ADF	42.0	3.9	5.1	
Lignin	6.9	1.6	0.8	
Ash	6.8	1.6	7.3	

1 Treatment diets were balanced with increasing forage-to-concentrate ratios, with the control diet being only hay, and remaining diets containing an increasing combination of cracked corn and soybean meal.

2 CG = corn grain; SBM = soybean meal.

Chopped hay samples were collected daily, then combined to make a composite representative of each period. Samples of both corn grain and soybean meal were taken from every bag used during the trial and composited by feed before analysis. After feed samples were obtained, they were stored at −20°C. Feed samples were sent to Cumberland Valley Analytical Services (Waynesboro, PA) for proximate analysis. The analysis conducted included DM (Goering and Van Soest, 1970; Shreve et al., 2006), nitrogen (AOAC method 990.03, AOAC International, 2005; Leco FP-528 Nitrogen Combustion Analyzer, Leco Corp., St. Joseph, MI), NDF (Van Soest et al., 1991), lignin (Goering and Van Soest, 1970), ADF (AOAC method 973.18; Horwitz and Latimer, 2000), ash (AOAC method 942.05; Horwitz and Latimer, 2000), and mineral contents (AOAC method 985.01; Horwitz and Latimer, 2000).

Each animal was fitted with a rumen fluid sampling line before the start of each sampling period. The sampling devices consisted of plastic tubing terminating in an 8.5 cm × 8.5 cm × 3.2 cm polypropylene knitted mesh scourer (Lola Products, Hackensack, NJ), weighted with 4 to 6 steel nuts. The end of each sampling line was placed below the rumen fiber mat. The tubing was allowed to extend out of the cannula and was connected to a Leur lock syringe to facilitate sampling. Samples of approximately 50 mL were obtained at each sampling time and were aliquoted into three 15-mL centrifuge tubes before being stored at −20°C. The Orion Star 2115101 Dual Star pH/ISE Benchtop Meter (Thermo Fisher Scientific, Waltham, MA) was used to measure pH of rumen fluid samples before freezing utilizing an Orion pH electrode probe (Thermo Fisher Scientific).

Volatile fatty acid concentrations were analyzed using gas chromatography. Thawed rumen fluid samples (1 mL) were acidified with 0.17 mL of metaphosphoric acid (25%, wt/vol) and 0.13 mL of internal standard (5 mmol, 4-methyl-valeric acid), vortexed, and allowed to rest for 30 min at 4°C. Samples were then centrifuged at 3,000 × *g* for 15 min. The supernatant was collected, placed in autosampler vials, and stored at −20°C until further analysis. The concentrations of total and individual VFA were determined using a 6,890 N Network GC System Gas Chromatograph (Agilent Technologies, Santa Clara, CA) equipped with a Quadrex 007–10 Series (Quadrex Corp., New Haven, CT) bonded phase fused silica capillary column. Nitrogen was used as the carrier gas with a flow rate of 1 mL/min. The temperature of the column was set at 60°C held for 2 min, increased to 100°C (10°C/min), increased to 200°C (20°C/min), and held for 5 min. One microliter of the sample was injected at split 1:30, at a temperature of 230°C. To avoid carryover effects and maintain consistent conditions, a sample of distilled water was injected between each sample. Each run lasted 18 min, which allowed for the separation of acetate (retention time: 1.6 min), propionate (3.0 min), isobutyrate (5.2 min), butyrate (12.9 min), and valerate (16.1 min). Commercial standards (Sigma-Aldrich, St. Louis, MO) of acetic (45997), propionic (94425), iso-butyric (46935), butyric (19215), iso-valeric (78651), valeric (75054), and caproic (21529) acids were used as external standards for peak identification. The molar concentrations of VFA were identified based on the single point internal standard and calibration curve with external standards. The column used for VFA determination in this analysis was not able to separate 2-methyl-butyric acid from isovaleric acid, and as such, the values labeled isovalerate in this work are most appropriately interpreted as isovalerate plus 2-methyl-butyrate.

Statistical analyses were performed using R version 4.1.2 (R Core Team, 2021) using the lme4 and lmerTest packages (Bates et al., 2014). To analyze the effect of dietary conditions on the concentrations and molar proportions of individual VFA, mean VFA concentrations were determined for each diet as the average across available time-series samples. These averages were then analyzed using a linear mixed effects model with fixed effects for forage:concentrate ratio (linear and quadratic) and random effects for animal and period. Analysis of variance was performed for each model to explore variable significance. Estimated marginal means were calculated using the emmeans package (Lenth and Lenth, 2018). Significance was declared at *P* < 0.05 and tendencies were described when 0.05 ≤ *P* < 0.10.

Before analyzing the pH measurements, these data were screened for erroneous measurements through visual and statistical exploration of data distributions. Based on these distributions, we omitted a single outlier (pH = 5.1) that exceeded 4.5 standard deviations below the mean and was greater than 1 standard deviation from the next lowest value. This omission resulted in 207 observations used for exploring the associations between rumen pH and VFA. To characterize this relationship, models were derived using linear mixed effects regression with fixed effects for measured pH (linear and quadratic) and random effects for animal and period. The capacity of the models to explain variation in VFA was evaluated using the residual error variance $\left(\hat{\sigma_{E}}\right).$ The models of VFA based on pH observations were compared with models derived with fixed effects for F:C ratio, time, and the interaction of treatment and time, to contextualize variability explained by the pH measurements compared with variability attributable to feed type and time postfeeding. We hypothesized that the pH measurements would capture more individual animal variability and provide better capacity to explain variability in rumen VFA measurements than the models based on diet and time.

Short-term disruption in F:C ratio resulted in differences in individual and total VFA concentrations (Table 2), with most concentrations responding quadratically to the increase in concentrate proportion of the diet. This response was expected because concentrate inclusion elevated the availability of energy and nutrients (Table 1) for microbial fermentation processes, supporting enhanced concentrations of VFA (Manoukian et al., 2021) and branched-chain VFA (Syamsi et al., 2019). Although the relationships linking F:C ratio to VFA concentrations are well established (Bergman, 1990; Dijkstra, 1994), most of the body of work on these relationships relies on long-term feeding, and comparison of samples obtained after adaptation to diets. Confirmation that VFA concentration changes are detectable within the short term supports their use as a potential target for incorporation into precision feeding algorithms to better represent individual animal fermentation status. Despite this opportunity, some changes expected to be observed with differing F:C ratio were not apparent after these short-term dietary disruptions. For example, in the present analysis molar proportions of VFA were not affected by linear or quadratic F:C ratio effects; however, higher concentrate inclusion is typically associated with decreased acetate to propionate ratio (Wang et al., 2016). When designing future precision feeding strategies, these response timelines should be carefully considered because delays in physiological responses may need to be expressly accounted for to ensure optimal productivity and health outcomes.

**Table 2:** Estimated mean VFA concentrations or molar proportions (MP) for diets varying in forage-to-concentrate ratio^1^

Item	Acetate, m*M*(SEM)	Propionate, m*M*(SEM)	Butyrate, m*M*(SEM)	Isobutyrate, m*M*(SEM)	Isovalerate, m*M*(SEM)	Total VFA, m*M*(SEM)	Acetate, MP (SEM)	Propionate, MP (SEM)	Butyrate, MP (SEM)	Isobutyrate, MP (SEM)	Isovalerate, MP (SEM)
Treatment^1^ (forage:concentrate)											
100:0	47.5 (4.1)	5.19 (0.33)	3.64 (0.31)	1.11 (0.07)	0.59 (0.05)	58.5 (4.9)	80.4 (0.8)	9.38 (0.32)	6.44 (0.32)	2.11 (0.12)	1.05 (0.06)
85:15	47.5 (3.4)	5.41 (0.27)	4.03 (0.27)	1.10 (0.06)	0.60 (0.04)	59.1 (4.0)	79.9 (0.6)	9.48 (0.26)	6.91 (0.25)	2.02 (0.09)	1.03 (0.05)
70:30	57.0 (3.4)	6.63 (0.27)	4.89 (0.27)	1.21 (0.06)	0.70 (0.04)	71.0 (4.0)	79.9 (0.6)	9.63 (0.26)	7.01 (0.25)	1.86 (0.09)	0.99 (0.05)
55:45	76.2 (4.1)	8.86 (0.33)	6.21 (0.31)	1.44 (0.07)	0.88 (0.05)	94.1 (4.9)	80.4 (0.7)	9.83 (0.32)	6.76 (0.32)	1.63 (0.12)	0.95 (0.07)
*P*-value^2^											
Linear	0.016	0.003	0.033	0.066	0.053	0.014	0.501	0.79	0.305	0.409	0.786
Quadratic	0.032	0.011	0.092	0.108	0.098	0.031	0.499	0.87	0.276	0.563	0.858

1 Treatment diets formulated with increasing forage-to-concentrate ratios, with the control diet being only hay (100:0), and remaining diets containing an increasing amount of concentrate (15%, 30%, 45%). Concentrate included cracked corn and soybean meal.

2 Significance was declared at *P* < 0.05, and statistical tendency was declared when 0.05 ≤ *P* < 0.10.

Significant linear and quadratic relationships linking measured pH to VFA concentrations were identified for most VFA; however, the $\hat{\sigma_{E}}$ of these models ranged from 21% to 38%, suggesting substantial variation in VFA concentration was not explained by the linear and quadratic relationships with pH (“pH-based model,” Table 3). Based on this poor performance, residuals analysis was performed on the predictions of the pH-based VFA models, regressing the residuals on F:C ratio, to explore whether the pH relationships derived were equally adequate among diets (“residuals analysis,” Table 3). In all cases, the residual VFA from the pH-based models were significantly affected by F:C ratio. The estimated marginal means from the regression of pH-based VFA prediction residuals against F:C ratio revealed that models consistently underpredicted VFA concentrations at low F:C ratios. In the example case of acetate, the pH-based model underpredicted acetate concentration of the 45% concentrate ration by 17.2 m*M* and overpredicted acetate concentration of the 100% forage ration by 6.4 m*M*. This residuals analysis suggests that the relationships between pH and VFA are inconsistent across diets, making pH a poor candidate for VFA sensing in precision feeding applications.

**Table 3:** Comparison and evaluation of strategies to predict VFA concentrations from pH or from diet and time postfeeding^1^

Item	Acetate, m*M*	Propionate, m*M*	Butyrate, m*M*	Isobutyrate, m*M*	Isovalerate, m*M*	Total VFA, m*M*
pH-based model						
*P*-value^2^						
Linear pH	0.032	0.015	0.028	0.241	0.322	0.030
Quadratic pH	0.037	0.019	0.036	0.253	0.343	0.034
Fit statistic						
$\hat{\sigma_{E}},$ m*M*	21.6	2.07	1.49	0.255	0.195	25.5
$\hat{\sigma_{E}},$ %	37.8	31.7	32.0	21.0	29.6	36.1
Residuals analysis for pH-based model predictions vs. F:C ratio						
Estimated marginal means (SEM)						
100:0	−6.44 (3.1)	−0.95 (0.28)	−0.724 (0.23)	−0.23 (0.05)	−0.23 (0.04)	−7.66 (3.7)
85:15	−10.8 (3.1)	−1.1 (0.28)	−0.528 (0.23)	−0.22 (0.05)	−0.21 (0.04)	−12.4 (3.7)
70:30	−0.44 (3.1)	−0.11 (0.28)	−0.074 (0.23)	−0.16 (0.05)	−0.15 (0.04)	0.16 (3.7)
55:45	17.2 (3.1)	1.97 (0.28)	1.34 (0.23)	0.13 (0.05)	0.065 (0.04)	19.9 (3.7)
*P*-value^2^	<0.001	<0.001	<0.001	<0.001	<0.001	<0.001
F:C ratio						
Diet-based model						
*P*-value^2^						
F:C ratio	0.060	0.077	0.069	0.033	0.001	0.059
Time	0.011	0.002	0.018	<0.001	<0.001	0.008
F:C ratio × time	0.269	0.001	0.008	0.236	0.566	0.173
Fit statistic						
$\hat{\sigma_{E}},$ m*M*	19.0	1.65	1.23	0.22	0.16	22.1
$\hat{\sigma_{E}},$ %	33.3	25.3	26.4	18.4	24.2	31.3

1 pH-based models used point-in-time measurements of pH and VFA to estimate VFA concentrations based on linear and quadratic relationships with measured pH. $\hat{\sigma_{E}},$ = residual error standard deviation expressed in m*M* or %; F:C ratio = forage-to-concentrate ratio used in treatment diets.

2 Significance was declared at *P* < 0.05, and statistical tendency was declared when 0.05 ≤ *P* < 0.10.

Models expressing VFA as a function of treatment, time, and the treatment by time interaction had $\hat{\sigma_{E}}$ ranging from 22.1% to 33.3% (i.e., “diet-based model”; Table 3). Diet-driven models were able to reduce $\hat{\sigma_{E}}$ by 3.4 to 6.4 percentage units compared with the pH measurement models, suggesting prediction of VFA from diet had greater capacity to explain variation than did prediction from pH. This improvement in explained variation in VFA is consistent with the residuals analysis on the pH-based predictions, further reinforcing the idea that pH measurements are not adequate, diet-independent predictors of VFA. The limited ability to characterize VFA concentrations from pH measurements may be due to the ubiquity of pH as a response within the rumen. Several individual- and herd-level factors, including milk yield, stage of lactation, diet, and age, can drive ruminal pH changes, meaning that pH is somewhat nonspecific as a metric of fermentation outcomes (Geishauser et al., 2012).

Currently no commercially sensing technologies are available that accurately determine ruminal VFA concentrations in real time; however, due to the physiological links between pH and VFA, we hypothesized that leveraging pH measurements for characterizing VFA concentrations may be a feasible strategy. Although pH measurements were significantly related to VFA concentrations, models derived from these data had large $\hat{\sigma_{E}},$ and residuals patterned significantly against F:C ratio. Models predicting VFA from F:C ratio, time, and their interaction resulted in lower $\hat{\sigma_{E}},$ supporting the conclusion that relationships between pH and VFA are not independent of diet conditions. These findings suggest that pH alone is not a reliable method for sensing ruminal VFA concentrations in response to short-term dietary treatments. Future work should explore the pH/VFA relationship after longer feeding periods, or explore strategies to use pH measurements and dietary characteristics for VFA prediction.

## References

[bib1] AOAC International (2005).

[bib2] Bates D., Maechler M., Bolker B., Walker S., Christensen R.H., Singmann H., Dai B., Scheipl F., Grothendieck G., Green P. (2014). Package ‘lme4’. http://lme4.r-forge.r-project.org.

[bib3] Bergman E.N. (1990). Energy contributions of volatile fatty acids from the gastrointestinal tract in various species. Physiol. Rev..

[bib4] Dijkstra J. (1994). Production and absorption of volatile fatty acids in the rumen. Livest. Prod. Sci..

[bib5] Dijkstra J., Ellis J.L., Kebreab E., Strathe A.B., López S., France J., Bannink A. (2012). Ruminal pH regulation and nutritional consequences of low pH. Anim. Feed Sci. Technol..

[bib6] Enemark J.M.D. (2008). The monitoring, prevention and treatment of sub-acute ruminal acidosis (SARA): A review. Vet. J..

[bib7] Geishauser T., Linhart N., Neidl A., Reimann A. (2012). Factors associated with ruminal pH at herd level. J. Dairy Sci..

[bib8] Goering H.K., Van Soest P.J. (1970).

[bib9] Horwitz W., Latimer G.W. (2000).

[bib10] Lampila M. (1955). Preliminary studies on the variations of pH and volatile fatty acid concentration of the rumen contents of the cow. Agric. Food Sci..

[bib11] Lenth R., Lenth M.R. (2018). Package ‘lsmeans.’. Am. Stat..

[bib12] Manoukian M., DelCurto T., Kluth J., Carlisle T., Davis N., Nack M., Wyffels S., Scheaffer A., Van Emon M. (2021). Impacts of rumen degradable or undegradable protein supplementation with or without salt on nutrient digestion, and VFA concentrations. Animals (Basel).

[bib13] Monroe C., Perkins A. (1939). A study of the pH values of the ingesta of the bovine rumen. J. Dairy Sci..

[bib14] NASEM. (National Academies of Sciences, Engineering, and Medicine) (2021).

[bib15] Penner G.B. (2014). Proc. 25th Florida Ruminant Nutrition Symposium.

[bib16] Penner G.B., Beauchemin K.A., Mutsvangwa T. (2006). An evaluation of the accuracy and precision of a stand-alone submersible continuous ruminal pH measurement system. J. Dairy Sci..

[bib17] Price T.P., Souza V.C., Liebe D.M., Elett M.D., Davis T.C., Gleason C.B., Daniels K.M., White R.R. (2021). Short-term adaptation of dairy cattle production parameters to individualized changes in dietary top dress. Animals (Basel).

[bib18] Sato S. (2016). Pathophysiological evaluation of subacute ruminal acidosis (SARA) by continuous ruminal pH monitoring. Anim. Sci. J..

[bib19] Sato S., Kimura A., Anan T., Yamagishi N., Okada K., Mizuguchi H., Ito K. (2012). A radio transmission pH measurement system for continuous evaluation of fluid pH in the rumen of cows. Vet. Res. Commun..

[bib20] Shreve B., Thiex N., Wolf M. (2006).

[bib21] Smith V.R. (1941). In vivo studies of hydrogen ion concentrations in the rumen of the dairy cow. J. Dairy Sci..

[bib22] Souza V.C., White R.R. (2021). Variation in urea kinetics associated with ruminant species, dietary characteristics, and ruminal fermentation: A meta-analysis. J. Dairy Sci..

[bib23] Syamsi A.N., Waldi L., Widodo H.S., Harwanto (2019). Branched chain volatile fatty acids profile of rumen fluids supplemented by different meal protein sources and protein-energy synchronization index. IOP Conf. Ser. Earth Environ. Sci..

[bib24] Van Soest P.V., Robertson J.B., Lewis B.A. (1991). Methods for dietary fiber, neutral detergent fiber, and nonstarch polysaccharides in relation to animal nutrition. J. Dairy Sci..

[bib25] Wang S., Wang W., Tan Z. (2016). Effects of dietary starch types on rumen fermentation and blood profile in goats. Czech J. Anim. Sci..

